# Reduction of Bacteria in Relation to Feeding Regimes When Treating Aquaculture Waste in Fly Larvae Composting

**DOI:** 10.3389/fmicb.2020.01616

**Published:** 2020-07-16

**Authors:** Ivã Guidini Lopes, Cecilia Lalander, Rose Meire Vidotti, Björn Vinnerås

**Affiliations:** ^1^Aquaculture Center of Unesp (Caunesp), São Paulo State University (UNESP), São Paulo, Brazil; ^2^Department of Energy and Technology, Swedish University of Agricultural Sciences, Uppsala, Sweden; ^3^São Paulo Agency of Agribusiness and Technology (APTA), Polo Regional Centro-Norte, Pindorama, Brazil

**Keywords:** BSF, ecotechnology, hygienization, *Escherichia coli*, Salmonella Senftenberg, *S.* Typhimurium, *S.* Typhi, *S.* Dublin

## Abstract

This study evaluated the impact of feeding regimes on process performance and inactivation of microorganisms during treatment of aquaculture waste with black soldier fly (BSF) larvae. In three treatments (T1–T3), a blend of reclaimed bread and aquaculture waste was used as substrate for BSF larvae. In T1, the substrate was inoculated with four subtypes of *Salmonella* spp. and *Escherichia coli* (both at 1% *w*/*w*), and offered only once, at the beginning of the 14-day trial. In T2 and T3, the substrate was supplied on three different days, with contaminated substrate provided only the first event in T2 and in all three events in T3. Provision of a lump sum feeding (T1) proved unfavorable for larval growth and process efficiency, but did not affect the microbial reduction effect. The total reduction in *Salmonella* spp. was approximately 6 log_10_ in T1 and T2, and 3.3 log_10_ in T3, while the total reduction in *E. coli* was approximately 4 log_10_ in T1 and T2, and 1.9 log_10_ in T3. After removing the larvae, the treatment residues were re-inoculated with *Salmonella* spp. and *E. coli*. It was found that the inactivation in both organisms continued in all treatments that originally contained BSF larvae (T1–T3), suggesting that antimicrobial substances may have been secreted by BSF larvae or by its associated microbiota.

## Introduction

Global fisheries and aquaculture production exceeded 170 million tons in 2016. Of this, 47% was produced exclusively by aquaculture, a fast-growing industry providing high-quality animal protein worldwide (FAO, 2018). Generation of solid organic waste occurs throughout all production steps in aquaculture, from nurseries to fattening stages, at fish processing plants, research centers and up to the final consumer (Love et al., 2015; Lopes et al., 2019). Fish waste (e.g., whole carcasses and body parts) typically contains high nutrient loads, which can be detrimental to the environment if inadequately disposed, causing soil and water contamination (Erondu and Anyanwu, 2005). In addition, as this waste stream decomposes rapidly, its microbial communities multiply during decomposition, thus posing a risk of disease transmission, as these residues may contain pathogens (Ghaly et al., 2010; Sousa et al., 2014).

It is well established that fish carry a diverse community of microorganisms in their gut and skin (Leroi and Joffraud, 2011). These include autochthonous microbiota and pathogenic bacteria originating from the aquatic environment where the fish are reared and from inadequate processing (Mol and Tosun, 2011). Cross-contamination may also occur and compromise non-contaminated materials, e.g., when the same processing equipment is used for contaminated and non-contaminated fish (Ghaly et al., 2010). The different microorganisms present include Gram-positive bacteria such as *Enterococcus* spp. and *Clostridium* spp. (Leroi and Joffraud, 2011), and Gram-negative bacteria belonging to the Enterobacteriaceae family, such as *Escherichia coli* and *Salmonella* spp. (Morris et al., 1970; Marchaim et al., 2003). Under favorable conditions (e.g. moisture and temperature), fish spoilage proceeds from autolytic deterioration to microbial degradation within 2–5 days (Shawyer and Pizzali, 2003).

Sanitization of organic wastes can be achieved by various treatment methods, such as thermophilic composting (Soobhany et al., 2017) and anaerobic digestion (Grudziñsk et al., 2015). A novel treatment using larvae of the black soldier fly (BSF) (*Hermetia illucens* L.) (Diptera: Stratiomyidae) has also been shown to be promising in relation to the sanitization of different biodegradable waste streams. During treatment by BSF larvae, initial waste volumes can be reduced by up to 85% (wet basis), while generating two marketable products: a protein-rich larval biomass to be used in replacement of soybean and fishmeal in animal diets (Wang and Shelomi, 2017), and an organic treatment residue that can be used as a soil amendment (Setti et al., 2019), both representing more sustainable alternatives for the transition to a circular economy. Additionally, there is evidence that BSF larvae treatment of organic wastes have an impact on the concentration of selected microorganisms, thus improving its hygiene quality (Erickson et al., 2004; Choi et al., 2012; Lalander et al., 2013).

In a study examining the effect of BSF larvae treatment of chicken manure inoculated with *E. coli* and *S. enterica*, Erickson et al. (2004) observed that the presence of larvae accelerated inactivation of both bacteria. However, they also found that, while the observed reductions in microbial levels were significant, they were insufficient to ensure complete safety of the treated manure as a soil amendment. Similarly, Lalander et al. (2013) found significant reductions in *Salmonella* spp. when treating human feces with BSF larvae, but detected the pathogen in the gut of the larvae at the end of treatment. They recommended an additional processing step for the product to ensure its hygienic quality.

Although several studies have demonstrated the capacity of BSF larvae to inactivate microorganisms, the mechanism behind this inactivation remains poorly understood. Among several existing interactions between BSF larvae and the environment, two possible mechanisms for the inactivation have been suggested: (1) passage through the BSF larvae gut and associated exposure to low pH (Coluccio et al., 2008); and (2) secretion of antimicrobial substances, such as organic acids and peptides that bind to the bacterial cell wall, by BSF larvae (Choi et al., 2012; Park et al., 2014; Vogel et al., 2018). Other studies have demonstrated strong interactions between the medium and BSF larvae microbiota, with significant interferences related to biotic and abiotic factors that could lead to distinct microbiological responses in the treatment of wastes as a whole (Wynants et al., 2018; Jiang et al., 2019). The mechanism that contributes most to microorganism inactivation, and whether different feeding regimes generate different results in terms of inactivation, remain to be determined.

Different systems (batch or semi-batch feeding systems) for organic waste treatment using BSF larvae have produced promising results in terms of waste sanitization, in particular for two microorganisms: *E. coli*, an indicator microorganism of fecal contamination, and *Salmonella* spp., a zoonotic pathogen (Hasan et al., 2019). Erickson et al. (2004) observed the inactivation of these microorganisms in a batch-mode system using different manures as growth substrate, and observed significant reductions in the populations of both (1.5 – 5 log for *E. coli* and 0.5 – 4.0 log for *Salmonella* spp. at varying temperatures). In a continuous BSF larvae reactor to which substrate was added three times a week and small larvae twice a week, Lalander et al. (2015) obtained significant reductions in *Salmonella* spp., but observed small inactivation in thermotolerant coliforms, where *E. coli* is the dominant species (Hachich et al., 2012). These findings suggest that there could be an impact of treatment system, the time in the treatment at which contaminated waste is added and feeding regime, on microorganism inactivation in BSF larvae treatment.

The aims of this study were to investigate the impact of feeding regimes and time of bacterial contamination on inactivation of selected microorganisms, and assess whether the treatment residue had antimicrobial properties.

## Materials and Methods

### Materials

Reclaimed bread of different brands and nutritional composition was donated by a local distribution company in Uppsala, Sweden (Fazer^®^). The selected model substrate for aquaculture waste (*Oncorhynchus mykiss* carcasses) was supplied by a commercial fish farm (Nordic Trout^®^) located in Mora (Sweden). Upon collection, the bread was manually shredded and placed inside plastic bags, while the aquaculture waste was milled in a meat grinder, homogenized, and placed in plastic bags. Both materials were kept at −18°C until use.

In a pre-trial performed before the start of the experiment, it was observed that the pH of the substrate (aquaculture waste and bread) declined significantly (<4.0) after approximately 72 h. This confounded the aim of the study, as pathogen survival may be jeopardized by low pH conditions. In order to avoid this drop in pH, the bread was moistened with a phosphate buffer solution (pH 7.2; SVA, Sweden) prior to the beginning of the experiment, to a moisture content of approximately 65%.

The larvae used in the experiments were obtained from a colony that has been running continuously since 2015, located at the Swedish University of Agricultural Sciences (SLU, Uppsala). Newly hatched larvae were reared on a substrate containing chicken feed (Granngården Hönsfoder Start) 20% dry matter, DM) and larvae treatment residues (1:1 ratio), for approximately 7 days. Larvae (1.2 mg wet mean weight) were separated by sieving (1 mm mesh), and three batches of 100 larvae were counted and weighted for batch weight estimation.

The pathogens used in this experiment were four serotypes of the Gram-negative bacteria *Salmonella enterica* (*S.* Senftenberg, *S.* Typhimurium, *S.* Typhi, and *S.* Dublin), and *E. coli* ATCC 13706, a specific microorganism used as an indicator of fecal contamination. The Gram-positive bacteria *Enterococcus* spp. was also evaluated throughout the study. It was not inoculated into the substrate, but was already present in the fish carcasses, at an approximate concentration of 10^5^ CFU g^–1^. *E. coli* (unknown strain) was also present in the carcasses at low concentrations (10^3^ – 10^4^ CFU g^–1^) – which would be further diluted by the addition of bread – thus we chose to inoculate this bacteria in the substrate in order to begin with higher concentrations and more stable populations.

Bacterial inoculate solutions were prepared according to the following procedure: the selected bacterial strains were grown at 37°C for 2 h, at 200 revolutions per minute (rpm), in 5 mL nutrient broth (Oxoid AB, Sweden). This concentrated solution was diluted in 45 mL of nutrient broth and kept in the same conditions for 24 h. Finally, the bacterial solution was centrifuged at 4500 rpm for 15 min at 15°C, and the pellet was collected and dissolved in 50 mL of Tween buffer (buffered NaCl peptone water with Tween 80 at pH 7; SVA, Sweden). The acquired concentrations of *Salmonella* spp. and *E. coli* in these concentrated solutions were approximately 10^7^ and 10^6^ CFU g^–1^, respectively, being determined by plating different diluted solutions originated from the concentrated solutions. The inoculation was performed directly on the combined substrate, at a rate of 1% *w/w*, before it was added to the treatments.

### Experimental Set-Up and Sampling

**FIGURE 1:**
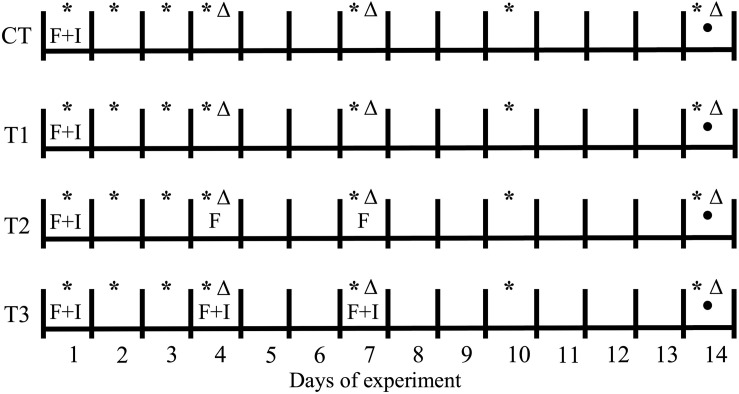
Schematic representation of the feeding and pathogen inoculation protocols throughout the first 14-day experiment with BSF larvae. CT, treatment without BSF larvae; F, feeding event; I, inoculation of pathogens; (*) pH measurement; (Δ) evaluation of total ammonium nitrogen (TAN) in the treatment residues; (⚫) total nitrogen (N_T_) measurement.

Two experiments were performed, aimed at: (1) determining the impact of feeding regime on BSF larvae treatment; and (2) investigating the inactivation potential of BSF larvae in the treatment of contaminated wastes, in order to evaluate the antimicrobial potential of the treatment residue, which serves as an indication of the presence of antimicrobial substances. The first experiment consisted of a control (CT) and three treatments (T1–T3), representing different feeding strategies. In T1, the feeding substrate was inoculated with four salmonella strains and *E. coli* and added once, on the first day of the experiment. In T2 and T3, the substrate was added three times, on day 1, day 4, and day 7, but was only inoculated on the first feeding (day 1) in T2, whereas the substrate in all three feedings was inoculated in T3. The control treatment followed the same protocol as in T1, but without addition of larvae, in order to determine the impact of these organisms (Figure 1).

The experimental units used in the first experiment were plastic containers (21 × 17 × 11 cm) covered with netting to prevent escaping of larvae. Three replicates per treatment were assembled. A total of 1000 larvae were placed inside each unit, giving an approximate density of 4 larvae cm^–2^, and a feeding rate of 0.25 g volatile solids (VS) larvae^–1^ was applied (80% aquaculture waste, 20% bread waste on wet basis). The pH of the treatment residues was measured on days 1–4, 7, 10, and 14, as was the total ammonium nitrogen (TAN) concentration. On day 14 of the experiment, the larvae were manually separated from treatment residues and one sample of approximately 30 g was collected per replicate (larvae and residue) for analysis of DM, VS, and total nitrogen (N_T_) content (Figure 1).

In the second experiment (called regrowth trial), all treatments considered in the first trial, including CT, were included. Triplicate, 5 g samples, of the treatment residues (materials remaining in the experimental units after the first experiment) from each replicate were collected using a sterile spoon, individually transferred to 50 mL Falcon tubes, and inoculated with the same concentrated solutions of *Salmonella* spp. and *E. coli* as were used in the first experiment, with an approximate concentration of 10^6^ CFU g^–1^ and 10^5^ CFU g^–1^, respectively. After 24, 72, and 120 h, the concentrations of these bacteria were assessed, as described ahead. Both experiments were conducted in a Class II microbiological laboratory. The mean temperature inside the laboratory was 28.8 ± 1.8°C throughout the experiments.

### Physico-Chemical Analysis

All samples of larvae and treatment residues (30 g) were dried at 105°C for 14 h for DM determination and combusted at 550°C for 6 h for total VS evaluation. For pH measurement, 5 g of treatment residues from each experimental unit were dissolved in 20 mL of deionized water and agitated in a vortex mixer for 2 min. After 1 h at room temperature, the pH was measured using a bench pH meter (InoLab pH level 1).

For N_T_ analysis, 0.5 g of sample was diluted in 15 mL of concentrated H_2_SO_4_ and brought to a “rolling boil” for approximately 20 min on a heating plate, and cooled to room temperature. A 1 mL sample from this mixture was diluted 50 times in deionized water, and the pH was neutralized to a range of 4–7, using a 10 M NaOH solution. A 10 mL sample of this solution was digested following the instructions of the analysis kit Spectroquant^®^ Crack-Set 20 (1.14963.0001), and the nitrate concentration was measured using a spectrophotometer at 340 nm, following the provider’s instructions of the kit Spectroquant^®^ Nitrate Test (1.09713.0002). For analysis of TAN concentrations in treatment residues, 1 g of sample was dissolved in 4 mL of deionized water, mixed in a vortex mixer for approximately 2 min, and the concentration was measured using a spectrophotometer at 640 nm, following the instructions of the provider of the analysis kit Spectroquant^®^ Ammonium Test (1.00683.0001).

### Microbiological Analysis

Microbial concentrations were assessed on a daily basis in the first experiment and on days 1, 3 and 5 in the second experiment, according to the following procedure: 5 g of treatment residues were dissolved in 45 mL of Tween buffer (this dilution was named 10^–1^), and serial dilutions were prepared from this concentrated solution after a 15 min resting period. A 100 μL volume of the selected dilution was spread on xylose lysine desoxycholate agar (XLD) with novobiocin (Oxoid AB, Sweden) for *Salmonella* spp. enumeration, and on chromocult coliform agar for *E. coli* enumeration, both incubated at 37°C for 24 h. A 100 μL volume was also spread on Slanetz-Bartley agar (Oxoid AB, Sweden) and incubated at 44°C for 48 h for *Enterococcus* spp. enumeration (only the first experiment). Three plates were prepared daily for each of the pathogens evaluated per replicate in the first experiment and on the three evaluation days in the regrowth experiment. In order to reduce the detection limits of *Salmonella* spp. and *E. coli* concentrations, a 200 μL volume of the concentrated solution (10^–1^) was spread on five plates for each sample and enumerated with a detection limit of 10 CFU mL^–1^.

### Calculations

DM losses and N volatilization were assessed by deducting the amount found in the residues and larvae at the end of the experimental period from the total amount added to the treatment. Survival rates were calculated in T1, T2, and T3, and process performance was evaluated based on material reduction, bioconversion ratio (percentage of substrate converted into insect biomass), and protein conversion ratio (proportion of added protein converted into larval protein), all presented in percentage.

The material reduction was calculated as:

(1) $$\mathrm{Material}\,\mathrm{reduction}=1-\frac{m_{\text{DM}\,\mathrm{res}}}{m_{\text{DM}\,\mathrm{sub}}}$$

where m_DMres_ and m_DMsub_ are dry mass of final residues and initial substrate, respectively.

The bioconversion ratio (BCR) on dry matter basis was calculated as:

(2) $$\mathrm{BCR}=\frac{m_{\text{DM}\,\mathrm{larvae}}}{m_{\text{DM}\,\mathrm{sub}}}\times 100$$

where m_DMlarvae_ is dry mass of larvae at the end of the experiment.

The protein conversion ratio (PCR) was calculated as:

ProteinCR

(3) $$=\frac{m_{\text{DM}\,\mathrm{larvae}}\times \%{\mathrm{Pr}}_{\mathrm{larvae}}}{m_{\text{DM}\,\mathrm{sub}}\times \%{\mathrm{Pr}}_{\mathrm{sub}}}\times 100$$

where %Pr_*larvae*_ and %Pr_*sub*_ are total percentage of crude protein (DM basis) in larvae and initial substrate, respectively.

A further three indices were used to assess pathogen inactivation: inactivation rate constant (*k*), which reveals the log_10_ reduction per time unit (log_10_ d^–1^ h^–1^); decimal reduction (D_90_), representing the time (days) needed for a 1 log_10_ (90%) reduction in the microorganisms initially present in the contaminated material; and total logarithmic pathogen reduction (ΔLogRed), which represents the total pathogen inactivation from beginning to the end of the experimental period.

Rate constant *k* was calculated as:

(4) $$k=\,\frac{\left(\log_{10}\,N_{t}-\log_{10}\,N_{0}\right)}{\left(N_{t}-N_{0}\right)}$$

where N_t_ and N_0_ are bacterial concentration at time *t* and at the beginning, respectively.

D_90_ was then calculated as:

(5) $$D_{90}=\left(\frac{-1}{k}\right)$$

Δ*LogRed* was calculated as:

(6) $$\Delta \,\mathrm{LogRed}=\log_{10}\,\left(\frac{{\mathrm{CM}}_{\mathrm{at}\,t=0}}{{\mathrm{CM}}_{\mathrm{at}.\mathrm{out}\,t=i}}\right)$$

where CMat_*t*=__0_ is the estimated initial concentration in treatment residues, and CMat.out_*t*=_*_*i*_* is the final concentration at time *i*.

### Statistical Analysis

Statistical analyses were performed using R software, version 3.5.3 (R Core Team, 2019), and GraphPad Prism, version 8.2.1. The assumptions of normality of error (Shapiro–Wilk’s test) and homoscedasticity of variance (Levene’s test) were verified for all process efficiency and pathogen inactivation data. One-way analysis of variance (ANOVA) with 95% confidence interval was performed to compare larval growth and process efficiency parameters, and the variables relating to pathogen inactivation. When significant differences were found, a Tukey *post hoc* test was performed at 5% significance level, to look for differences between treatments in the variables analyzed. Linear regressions were performed to assess the effect of time on pathogen inactivation.

## Results

### Process Efficiency

The substrate biomass reduction exceeded 65% (DM basis) in all treatments containing larvae, which was significantly higher than in the control treatment (CT) without larvae (Table 1). Larvae growth also differed significantly between treatments. After 14 days, larvae fed only once at the beginning of the trial (T1) were on average 35% lighter than larvae fed three times (T2 and T3). Hence, the bioconversion and protein conversion ratios were approximately 30 and 20% lower, respectively, in T1 than in T2 and T3. Larval survival and crude protein (%) did not vary significantly between treatments (Table 1). The difference in bacteria inoculation regime between T2 and T3 (where T3 received *Salmonella* spp. and *E. coli* at each feeding and T2 only at first feeding) did not affect the parameters evaluated. The larvae recovered at day 14 were all pre-pupae in all trials.

**TABLE 1:** Efficiency of treatment of aquaculture waste and bread using black soldier fly (BSF) larvae.

	**CT**	**T1**	**T2**	**T3**
Bioconversion ratio (%)	–	16.72.2^b^	24.21.5^a^	24.30.4^a^
Protein conversion ratio (%)	–	14.33.1^b^	19.21.8^a^	18.11.0^a^
Material reduction (%)	35.9 ± 5.6^b^	70.12.6^a^	65.61.9^a^	66.22.1^a^
Final weight (mg)	–	109.013.0^b^	165.023.9^a^	171.912.7^a^
Larval CP (%_DM_)	–	47.64.9	44.12.3	41.53.1
Survival (%)	–	86.12.7	93.610.7	90.15.7

*CP, larval crude protein; CT, wastes inoculated with pathogens without larvae; T1, larvae fed once with inoculated wastes; T2, larvae fed three times, with wastes inoculated only the first time; T3, larvae fed three times, with wastes inoculated all three times. Values presented are mean ± SD. Different letters row-wise indicate significant differences (*p* < 0.05) between treatments.*

The pH of the feeding substrate at the beginning of the experiment was around 6.8, while at the end of the experiment it varied from 6.1 (CT) to 6.8 (T1), being significantly higher in T1 in comparison to other treatments. DM loss was higher in T1, where a single batch of substrate was supplied on the first day, than in T2 and T3. In all treatments, the remaining residues were dry after 14 days, with a DM content of 80–88%. The N_T_ concentrations in the treatment residues were not significantly different between treatments with larvae, but N volatilization was significantly higher (51%) in T1 than in T2 and T3 (38%) (Table 2).

**TABLE 2:** Physico-chemical characteristics of the treatment residues obtained when black soldier fly (BSF) larvae were fed aquaculture waste and bread, and losses of dry matter and nitrogen after 14 days of treatment.

	**CT**	**T1**	**T2**	**T3**
Residue DM (%)	88.41.3^a^	83.22.0^b^	79.12.3^b^	82.20.4^b^
Residue VS (%)	94.50.9^a^	91.41.2^ab^	89.71.7^b^	89.60.8^b^
DM loss (%)	35.95.6^b^	53.40.5^a^	41.42.4^b^	41.91.9^b^
N_T_ (g kg^−1^)	ND	24.31.3	24.40.3	26.01.7
N volatilization (%)	ND	51.31.3^a^	38.81.6^b^	37.90.9^b^
Final TAN (mM kg^1^)	11.32.5	12.91.9	13.16.8	12.33.8
Initial pH	6.70.1	6.80.1	6.80.0	6.90.1
Final pH	6.10.2^c^	6.80.1^a^	6.50.1^b^	6.40.1^b^

*DM, dry matter; VS, volatile solids; N_*T*_, total nitrogen; TAN, total ammonium nitrogen; ND, not determined; CT, wastes inoculated with pathogens without larvae; T1, larvae fed once with inoculated wastes; T2, larvae fed three times, with wastes inoculated only the first time; T3, larvae fed three times, with wastes inoculated all three times. Values presented are mean ± SD. Different letters row-wise indicate significant differences (*p* < 0.05) between treatments.*

**FIGURE 2:**
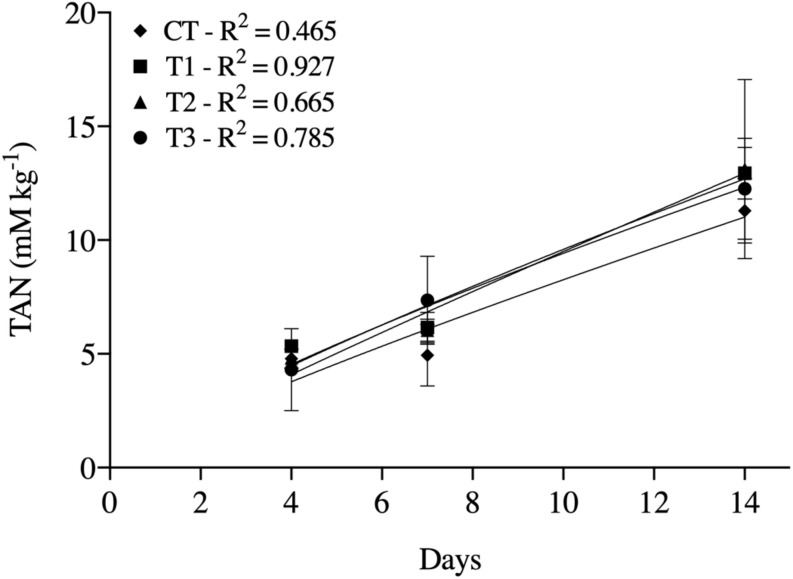
Total ammonium nitrogen (TAN) generated at days 4, 7, and 14 in treatment of aquaculture waste and bread with black soldier fly (BSF) larvae. CT, wastes inoculated with pathogens without larvae; T1, larvae fed once with inoculated wastes; T2, larvae fed three times, with wastes inoculated only the first time; T3, larvae fed three times, with wastes inoculated all three times. *R*^2^ values are coefficient of determination of the regression lines fitted for each treatment.

The TAN concentrations increased with time in all treatments. Significant differences between treatments were found only at day 14, when treatments containing BSF larvae showed higher TAN concentrations than CT (Figure 2).

### Pathogen Inactivation

**FIGURE 3:**
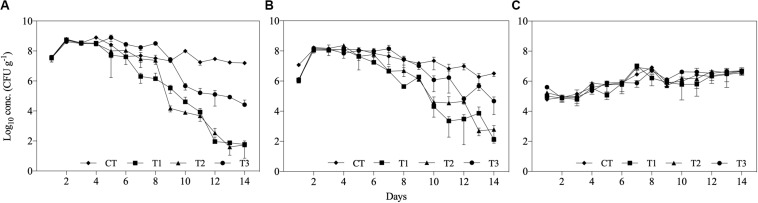
Log_10_ concentrations (CFU g^– 1^) of **(A)** *Salmonella* spp., **(B)** *Escherichia coli*, and **(C)** *Enterococcus* spp. over 14 days of treatment of aquaculture waste and bread treated with black soldier fly (BSF) larvae. CT, wastes inoculated with pathogens without larvae; T1, larvae fed once with inoculated wastes; T2, larvae fed three times, with wastes inoculated only the first time; T3, larvae fed three times, with waste inoculated all three times.

The concentrations of *Salmonella* spp. and *E. coli* were significantly reduced in all treatments after 14 days, while *Enterococcus* spp. concentration was not reduced in any of the treatments. Additionally, the differences between feeding regimes resulted in differences in microbial reduction patterns (Figure 3). The initial concentration of *Salmonella* spp. and *E. coli* in the inoculated substrate was approximately 10^7^ and 10^6^ CFU g^–1^, respectively, in all treatments. At the first day post-inoculation, increases of approximately 1 log_10_ were found for both bacteria in all treatments, while *Enterococcus* spp. populations increased by 1 log_10_ during the 14 days of the experiment (Figure 3).

The reduction in both *Salmonella* spp. and *E. coli* populations started within approximately 1 week of treatment and continued until the end of the experiment (Figure 3). In T3, where inoculated substrate was added three times, the concentrations of the evaluated microorganisms increased on subsequent days. However, the reduction in *Salmonella* spp. and *E.* coli in T3 at the end of the experiment was approximately 2.6 log_10_ and 2.2 log_10_ lower, respectively, than in the other treatments containing BSF larvae (Figure 3).

The reduction of *Salmonella* spp. was 5.8 log_10_ in T1, 6.2 log_10_ in T2, and 3.4 log_10_ in T3, while the reduction in the control (CT) with no larvae was 0.4 log_10_ (Table 3). The reduction rate (*k*; log_10_ CFU g^–1^ day^–1^) was similar over time for both *Salmonella* spp. and *E. coli* in T1 and T2, but significantly faster than in the control (Table 3). Similarly, the decimal reduction (D_90_) calculations revealed that the required time to reduce *Salmonella* spp. and *E. coli* populations by 90% (1 log_10_) was 2.4 days and 3.5–4.5 days in T1 and T2, respectively.

**TABLE 3:** Values of the reduction constant (*k, log_10_ CFU g^–1^ day^–1^*), decimal reduction (D_90_, days), mean log_10_ reduction (Δlog_10_Red), and *p-*value for different microorganisms in black soldier fly (BSF) larvae treatment of aquaculture waste and bread.

	***Salmonella* spp.**				***E. coli***				***Enterococcus* spp.**			
	***k***	**D_90_**	**Δlog_10_Red**	***p* for time effect**	***K***	**D_90_**	**Δlog_10_Red**	***p* for time effect**	***k***	**D_90_**	**Δlog_10_Red**	***p* for time effect**
T1	−0.412^a^	2.4^c^	5.75^a^	***	−0.281^a^	3.5^b^	3.97^a^	***	0.117	−8.5	−1.63	*
T2	−0.413^a^	2.4^c^	6.22^a^	***	−0.229^a^	4.4^b^	3.32^ab^	***	0.102	−9.8	−1.41	**
T3	−0.222^b^	4.5^b^	3.28^b^	***	−0.102^b^	9.7^c^	1.87^bc^	*	0.077	−12.9	−1.07	**
CT	−0.027^c^	36.4^a^	0.34^c^	**	−0.040^c^	24.8^a^	0.59^c^	**	0.126	−7.9	−1.51	*
T3^‡^	−0.291	3.4	4.25	***	−0.198	4.4	3.14	**	–	–	–	–

CT, wastes inoculated with pathogens without larvae; T1, larvae fed once with inoculated wastes; T2, larvae fed three times, with wastes inoculated only the first time; T3, larvae fed three times, with wastes inoculated all three times. Different letters column-wise indicate significant differences (*p* < 0.05) for *k*, D_90_ and Δlog_10_Red values. ^‡^Recalculated indexes for T3, considering the period between day 8 (1 day post final feeding) and day 14 (end of experiment); * *p* < 0.01; ** *p* > 0.001; *** *p* < 0.0001.

Based on the D_90_ and *k* values obtained for T3, a 90% reduction in both *Salmonella* spp. and *E. coli* from the first to last day of experiment took twice as long as in T1 and T2. Since T3 received inoculated substrate again on days 4 and 7, additional calculations of D_90_ and *k* were performed to assess the inactivation potential of this particular feeding regime. The D_90_ value from the day post final feeding (day 8) to the final day (day 14) for *Salmonella* spp. and *E. coli* was found to be lower than the value based on the concentrations found on days 1 and 14 of the experiment. Similarly, the *k* was found to be lower, 0.291 and 0.198 log_10_ CFU g^–1^ day^–1^ for *Salmonella* spp. and *E. coli*, respectively (Table 3).

### Regrowth Trial

The regrowth of microorganisms in the treatment residues (without larvae) in all treatments was evaluated for the four *Salmonella* strains and *E. coli*. The initial achieved concentration of *Salmonella* spp. and *E. coli* was approximately 10^6^ and 10^5^ CFU g^–1^, respectively. After 3 days, no reduction was observed for neither of these microorganisms. However, after 5 days, a reduction of approximately 2.5 log_10_ was observed in the treatments that previously had larvae (T1, T2, and T3), while the concentration remained unchanged in the control that had no had BSF larvae (CT). The total reduction in *E. coli* was similar to that in *Salmonella* spp. on residues from T1 and T2, as revealed by the Δlog_10_Red and *k* values after 5 days of the experiment. However, the total reduction on residues from T3 was significantly lower for *E. coli* (total reduction of 1.8 log_10_), while an increased concentration was found for the control treatment residue after 5 days (Table 4).

**TABLE 4:** Concentrations (log_10_) of *Salmonella* spp. and *Escherichia coli* in treatment residues derived from black soldier fly (BSF) larvae treatment of aquaculture waste and bread, at the first (**In**) and fifth (**Out**) days of the regrowth trial, and values of the reduction constant (*k, log_10_ CFU g^–1^ day^–1^*) and the mean log_10_ reduction (Δlog_10_Red) achieved.

	***Salmonella* spp. (log_10_)**				***E. coli* (log_10_)**			
	**In**	**Out**	**Δlog_10_Red**	***k***	**In**	**Out**	**Δlog_10_Red**	***k***
T1	6.76	4.14^b^	2.61^a^	−0.186^a^	5.80	3.44^c^	2.37^a^	−0.169^a^
T2	6.69	4.09^b^	2.60^a^	−0.185^a^	5.86	3.23^c^	2.63^a^	−0.187^a^
T3	6.87	4.56^b^	2.31^a^	−0.165^a^	6.05	4.22^b^	1.84^b^	−0.131^a^
CT	7.04	7.11^a^	−0.07^b^	0.005^b^	5.59	6.35^a^	−0.76^c^	0.054^b^

*CT, wastes inoculated with pathogens without larvae; T1, larvae fed once with inoculated wastes; T2, larvae fed three times, with wastes inoculated only the first time; T3, larvae fed three times, with wastes inoculated all three times different letters column-wise indicate significant differences (*p* < 0.05).*

## Discussion

### Efficiency of BSF Larvae Treatment in Different Feeding Regimes

The proportion between aquaculture and bread waste was adopted based on the results obtained by Lopes et al. (2020), whom observed that the addition of more than 25% of aquaculture waste to bread makes the treatment of this waste unfeasible. Supplying aquaculture-bread wastes as feeding substrates following different protocols (T1–T3) resulted in differences in growth and bioconversion ratios of BSF larvae, but did not affect survival of the larvae. Larvae in treatments T2 and T3 gained more weight than those in T1, which resulted in lower bioconversion ratio and protein conversion ratio (DM basis) in T3. However, the substrate biomass reduction was similar (∼65%) in all treatments containing larvae, indicating that more of the material was microbially degraded with only one substrate supply (T1), and that BSF larvae more efficiently converted a larger proportion of the substrate when three feedings were performed (T2 and T3).

Physico-chemical changes during fish waste microbial spoilage have been shown to result in lipid oxidation and protein degradation, through which N is lost by volatilization (Ghaly et al., 2010). Our results support this hypothesis, as N volatilization was higher in T1 than in T2 and T3 (Table 2). Therefore, the slower BSF larvae growth in T1 could have been caused by nutrient imbalance in the degraded substrate, or by increased bacterial respiration in the substrate. In contrast, Banks et al. (2014) observed larger final body weight, but slower growth, of BSF larvae fed feces substrate in a lump sum rather than every two days. They concluded that aging feces had lower nutritional quality than fresh feces, and therefore the BSF larvae consumed larger amounts of the substrate in order to compensate for this deficiency. Based on the obtained results, the treatment of aquaculture waste is better performed when three batches of the substrate is supplied instead of only once.

### Inactivation of Microorganisms

*Escherichia coli* is one of the most commonly used indicator organisms for fecal contamination (Carlos et al., 2010), and it occurs naturally in decaying carcasses of different fish species (Leroi and Joffraud, 2011), in accordance with the findings in this study. *Salmonella* spp., on the other hand, is a zoonotic bacteria that can infect most animals (Hasan et al., 2019) and several international standards demand absence of this microorganism in consumable products by animals and humans. It may also be found in decaying carcasses (Morris et al., 1970), however, this microorganism was not naturally observed in the carcasses used in this study. *Salmonella* spp. has been reported to be reduced in BSF larvae treatment, but the fate of *E. coli* is not consistent in previous studies. Therefore, they were chosen here as model microorganisms to evaluate the effect of treatment of aquaculture wastes.

Black soldier fly larvae treatment had strongest impact on *Salmonella* spp., with reductions of up to 6 log_10_ observed in T1 and T2. Based on larval growth and process efficiency parameters, *Salmonella* spp. inactivation was also affected by time of contamination: the treatment that received three contaminated feedings (T3) displayed a D_90_ of 4.5 days, in comparison with 2.4 days in T1 and T2, which resulted in lower total inactivation (∼3.3 log_10_ reduction) after 14 days in T3. A pronounced lag phase was observed in the effect on *Salmonella* spp. growth in T1-3, as the concentration started to be reduced within approximately 5–7 days after the beginning of the experiment (Figure 3A). Therefore, the difference in reduction potential of T1 and T2 compared with T3 seemed to be a consequence of adding contaminated substrate more than once (Figure 1).

A few studies have demonstrated that different pathogens in the Enterobacteriaceae family can be inactivated in BSF larvae treatment, using different feeding systems. For example, using a continuous fly reactor, in which BSF larvae were fed with inoculated substrate three times a week and prepupae was continuously collected, Lalander et al. (2015) observed significant reductions in *Salmonella* Typhimurium (>7 log_10_), beginning after 2 weeks of treatment and persisting for the remainder of their 9-week experiment, while the impact on thermotolerant coliforms was small. The authors hypothesized that a continuous system could improve the hygienic quality of the treated material compared with a batch-mode system, due to possible interactions of an established microbial and fungal community. As shown in the present study, it is not possible to adopt this BSF larvae treatment as a sole hygienization method, regardless of the system used, because, although some bacteria in the Enterobacteriaceae family are inactivated, the degree of inactivation is not sufficient to guarantee a completely safe end-product, regarding both larvae and treatment residues.

Gram-negative *E. coli* was inactivated over time up to 4 log_10_, while in the study of Lalander et al. (2015), a small inactivation was verified for thermotolerant coliforms. Erickson et al. (2004) verified only a small reduction in *E. coli* at 27°C (1.5 log_10_) and a more significant reduction (5 log_10_) at 32°C in one of the three feeding substrates (chicken manure) they evaluated. As reported by the authors of that study, such a strong inactivation of *E. coli* at 32°C in comparison to lower temperatures, might be due to increased concentration of uncharged ammonia in the manure, which is known to increase with temperature (Nordin et al., 2009). Zdybicka-Barabas et al. (2017) studied the immune response of BSF larvae challenged with Gram-positive (*Micrococcus luteus*) and Gram-negative (*E. coli*) bacteria, and verified that the larvae produce specific sets of antimicrobial peptides to fight those bacteria, while other microorganisms remained unaffected. In addition, Vogel et al. (2018) suggested that the immune response of BSF larvae is at least partly diet-dependent, and that protein-rich diets might lead to stronger immune defenses. The authors found stronger antimicrobial activity against *E. coli* in larvae fed proteins, in comparison to larvae fed a plant oil-rich diet or a lignin-rich diet. In this sense, it is possible to assume that *E. coli* inactivation in the present study may have partly occurred due to excreted antimicrobial peptides induced by the influence of diet, which was protein-rich as fish carcasses were included.

No reduction in the Gram-positive bacteria *Enterococcus* spp. was found in this study (Figure 3C). Different types of antimicrobial peptides have been isolated from the BSF larvae that displayed significant effects against different bacteria strains. Choi et al. (2012) found antimicrobial peptides in methanol extracts of BSF larvae that showed antimicrobial properties against members of the Enterobacteriaceae family, but with no impact on Gram-positive bacteria, while Shin and Park (2019) successfully isolated attacin from BSF larvae, a type of antimicrobial peptide that has shown antimicrobial properties to selected Gram-negative and Gram-positive bacteria. However, in this last case, the antimicrobial substances were isolated from the larvae after an immunization process in which the production of these substances were induced in the larvae. Considering that Lalander et al. (2013) found high levels of *Enterococcus* spp. in BSF larval gut, suggesting that this is not a threat to the larvae, it may be that the BSF larvae does not produce any antimicrobial substance effective against this microorganism as an immune response. Alternative, the conditions under which the experiment was undertaken or similar to the case for *E. coli* the substrate, did not induce the production of these peptides in this case. Furthermore, other properties may have an impact. *Enterococcus* spp. has specific characteristics in their cell wall that assures higher endurance, such as greater thickness, composition with more peptidoglycans in comparison to Gram-negative bacteria, and higher resistance to ruptures (Hancock et al., 2014).

Another impacting factor could have been the TAN concentrations, as they were higher in treatments containing larvae (T1-3) than in the control (CT) without larvae (Figure 2). In a study that evaluated *Salmonella* spp. inactivation using urea and ammonia, Fidjeland et al. (2016) obtained significant inactivation of this pathogen at different temperatures (5–32°C) using high concentrations of ammonia (>50 mM). In the present study, TAN concentrations did not exceed 13 mM kg^–1^, thus it is possible to assume that the effect of TAN on pathogen inactivation was possibly less relevant than the direct action of the BSF larvae. Similarly, although substrate temperature was not investigated directly in our study, it may have influenced microbial survival, as thoroughly demonstrated by Liu et al. (2008).

### Bacteria Regrowth

It is difficult to ensure that microorganisms are entirely eliminated when treating organic wastes, regardless of the method used, as bacterial regrowth may occur under appropriate conditions (e.g. moisture, temperature, and pH), even after the end of treatment (Soobhany et al., 2017). The degree of maturity of compost has been demonstrated by Elving et al. (2010) to affect bacterial regrowth; they found negative correlations between the growth potential of *Salmonella* Typhimurium and the degree of maturity of an organic compost. The maturity of a material in a composting process (e.g. thermophilic composting and BSF larvae treatment) can be determined either by the self-heating capacity of the material (Brinton et al., 1994) or by assessing the CO_2_ and NH_3_ emissions in the material, using simple commercially available tests (Changa et al., 2003). The treatment residue from fly larvae composting has been found to be that of raw compost (Lalander et al., 2018), so it is thus unlikely that the maturity of the residue was a main driver for continued inactivation in this study.

Although the impact of antimicrobial substances may play a key role in the inactivation of selected microorganism in a BSF larvae treatment, as discussed above, it is not clear whether the material has to pass through the larval gut to be in contact with these substances or whether they are excreted by the larvae. In the studies that have isolated antimicrobial substances, the substances were isolated from the larvae. Additionally, interactions between BSF larvae intrinsic bacteria and the medium bacteria may occur and further affect the dynamics of these microorganisms over time (Wynants et al., 2018; Jiang et al., 2019). The regrowth trial demonstrated that, after inoculating more microorganisms into the treatment residues, inactivation continued to occur even in the absence of BSF larvae, suggesting that antimicrobial substances were present in the treatment residue, or even that larval microbiota somehow affected microorganisms’ survival. The same patterns in inactivation of studied microorganisms that were seen in the composting phase was also seen in the regrowth trial, further supporting the hypothesis that antimicrobial substances excreted into the material (in this case treatment residue) contributed to the observed reduction in *Salmonella* spp. and *E. coli.*

## Conclusion

Feeding regimes were found to have an impact in BSF larvae composting of aquaculture waste. Larvae growth, bioconversion and protein conversion ratios was higher when the same amount of aquaculture waste was provided three times during the 14-days BSF larvae composting process as compared to when it was provided in a lump sum at the start of the treatment. Significant inactivation of *Salmonella* spp. (6 log) and *E. coli* (3.5 log) were achieved when the substrate was provided as a lump sum at the start, but when the substrate was inoculated three times these reductions were less pronounced. *Enterococcus* spp. was not affected by the treatment regardless of feeding regime. Even when larvae was removed from the treatment residues, both *Salmonella* spp. and *E. coli* continued to be inactivated in the material for 5 days, suggesting that there are antimicrobial substances present in the fly larvae composted material. Whether these substances originate from the larvae or from the larvae associated microbial community has to be investigated further. Regardless of the adopted feeding regimes, BSF larvae treatment cannot be considered a hygienization method, as it only improves the hygiene quality of the materials, thus it is recommended that both larval biomass and treatment residues undergo a post-treatment in order to ensure complete sanitization.

## Data Availability Statement

The datasets presented in this study can be found in online repositories. The names of the repository/repositories and accession number(s) can be found at: Mendeley Data repository – http://dx.doi.org/10.17632/n52h7d4gk2.1.

## Author Contributions

IL: conceptualization, investigation, formal analysis, writing the original draft, and visualization. CL: conceptualization, investigation, formal analysis, resources, supervision, funding acquisition, and writing – review and editing. RV: conceptualization and supervision. BV: conceptualization, resources, supervision, funding acquisition, and writing – review and editing. All the authors contributed to the article and approved the submitted version.

## Conflict of Interest

The authors declare that the research was conducted in the absence of any commercial or financial relationships that could be construed as a potential conflict of interest.
